# Periodontitis and alzheimer´s disease

**DOI:** 10.4317/medoral.23940

**Published:** 2020-07-23

**Authors:** Daniel Sansores-España, Arelly Carrillo-Avila, Samanta Melgar-Rodriguez, Jaime Díaz-Zuñiga, Victor Martínez-Aguilar

**Affiliations:** 1 DDS, Student. Specialization in Periodontics, School of Dentistry, Autonomous University of Yucatan, Mexico; 2 DDS, MINE, Professor. Specialization in Periodontics, School of Dentistry, Autonomous University of Yucatan, Mexico; 3 DDS, MSc. Professor. Department of Conservative Dentistry. Faculty of Dentistry, University of Chile, Chile; 4 DDS, MSc, PhD, Professor. Periodontal Biology Laboratory, School of Dentistry, University of Chile, Chile; 5 DDS, MIS, PhD, Professor. Specialization in Periodontics, School of Dentistry, Autonomous University of Yucatan, Mexico

## Abstract

**Background:**

Alzheimer's disease (AD), the main cause of dementia in the adult population, is characterized by a progressive loss of cognitive function. It is considered that neuroinflammation plays a fundamental role in its onset and progression. The bacteria present in the disbiotic microbiome generated during the course of periodontitis (PE) are capable of inducing a systemic inflammatory response, exacerbating the production of proinflammatory mediators that have the potential to spread to the systemic circulation.

**Material and Methods:**

A literature review was made using the databases Scielo, PubMed, EBSCO and key words "Alzheimer disease", "Periodontitis", "Neurodegeneration", "Inflammation mediators", "Elderly".

**Results:**

Several hypotheses point to similar pathophysiological pathways in the establishment of AD and PE, sharing cellular and molecular proinflammatory characteristics. In periodontitis, locally produced cytokines and pro-inflammatory products spread from the ulcerated periodontal pocket into the systemic circulation, or around the trigeminal nerve terminals, which allows the passage of bacteria or their products to the brain. This fact leads to the formation of plaques of amyloid peptide and intraneuronal neurofibrillar tangles (NFTs) that activate the glial cells producing a significant increase in proinflammatory cytokines in the affected regions that lead to a loss of neuronal synapses and neurodegeneration, contributing to the progression of AD.

**Conclusions:**

This review of the literature contributes to the understanding of the pathological pathways shared by both diseases such as oxidative damage and inflammation. There is not enough evidence to determine an association between this two pathologies, so it is considered necessary to conduct studies for determine if periodontitis is capable of inducing or exacerbating the neuroinflammation that will trigger AD.

** Key words:**Alzheimer´s disease, periodontitis, neurodegeneration, inflammatory mediators, elderly.

## Introduction

Alzheimer's disease (AD) is the most common form of dementia in elderly, constituting the main cause of dependence and disability in this population group. It appears more frequently in people over 65 years of age, though there is an affected group of younger age and is more prevalent in women than in men (1). According to the Worlds Alzheimer’s Report of 2018, there are 50 million people in the world living with dementia, of which two-thirds are affected by AD (2). As life expectancy increases, it is estimated that the incidence of AD increases to 152 million people by 2050, becoming a major health problem (1,3).

Early onset AD is believed to be extremely determined by genetic factors, while late or sporadic onset, which includes 95% of patients, resulting from the interaction of risk and environmental factors. Risk factors for AD onset include family history, education, high-fat diet, hypertension, diabetes, dyslipidemia, distress, insomnia, sedentary lifestyle, smoking, alcoholism, substance abuse, age, history of head trauma or susceptibility genes such as Amyloid Precursor Protein (APP), Beta-secretase (BACE) and Apolipoprotein Ɛ (APOƐ) (4).

Currently, several experimental models explain the pathology of AD with different onset and progression causes, however, it is considered that neuroinflammation plays a central role in the amyloid cascade hipothesis. The most accepted theory to explain the pathogenesis of AD is the amyloid cascade, where the neuroinflammation is the initial event conducting to the accumulation and aggregation of β-amyloid peptide (Aβ), followed by the formation of neurofibrillar tangles (NFT) formed by a hyperphosphorylated form of the Tau protein. The presence of Aβ induces hyperphosphorylation of Tau proteins and the formation of NFTs and thus, axonal dysfunction that generates higher accumulation of Aβ in synaptic spaces. The Aβ aggregates make up the senile plaques and, together with NFTs, constitute the histopathological features of AD (5-10).

It has been speculated that systemic infections may play an important role in establishing an inflammatory state in the central nervous system. In this regard, PE is one of the most common oral infections in the adult population, it is considered the second most common oral disease in humans and in older age groups a major cause of tooth loss (11,12). It derives from the establishment of a dysbiotic microbiome that is generated by keystone pathogens or pathobionts (13). These bacteria or its virulence factors can induce both direct damage to periodontal tissues or indirect damage by inducing the host's immune response, wich leads to the formation of subgingival pocket and evoking chronic inflammation in periodontal tissues and causes alveolar bone loss (14,15). This dysbiotic microbiome is capable of inducing a low-grade systemic inflammatory response by activating the host’s immune response and exacerbated production of pro-inflammatory mediators such as interleukin (IL)-1α, IL-1β, IL-6, tumor necrosis factor (TNF)-α, prostanoids and matrix metalloproteinases (MMPs) that have the potential to spread to the bloodstream (16). At the local level, the dysbiotic microbiome modulates the destruction of periodontal connective tissue and bone resorption (16). At the systemic level, it results in the host’s exposure to various cytokines and inflammatory mediators (17). The periodontal pathogens associated with periodontitis are rich in endotoxins and Lipopolysaccharide (LPS) that stimulate the activity of immune cells and the production of cytokines. Cytokines, such as IL-1β, IL-6 and TNF-α can spread to brain by bloodstream or by peripheric nerve terminals. Once in the brain, cytokines, bacteria or its virulence factors could, eventually, stimulate glial cells and induce neuroinflammation, which can contribute to the onset or progression of AD (18).

The aim of this article is to determine the pathological pathway through AD and PE can be associated, to allow an approach and higher knowledge about these two diseases so prevalent in eldery.

## Material and Methods

A literature review was made using the databases Scielo, PubMed, EBSCO, MEDLINE and DOAJ, for this purpose the keywords "Alzheimer's disease", "Periodontitis", "Neuroimmune mechanism", "Neurodegeneration", "Inflammation mediators" and "elderly" were used. The review was carried out in the period between December 2019-April 2020. 25 studies met the specific parameters and were included, among them bibliographic review studies, clinical trials, cases and controls, experimental studies and bibliographic meta-analysis.

## Results

- Alzheimer and periodontitis: links

**Figure 1:**
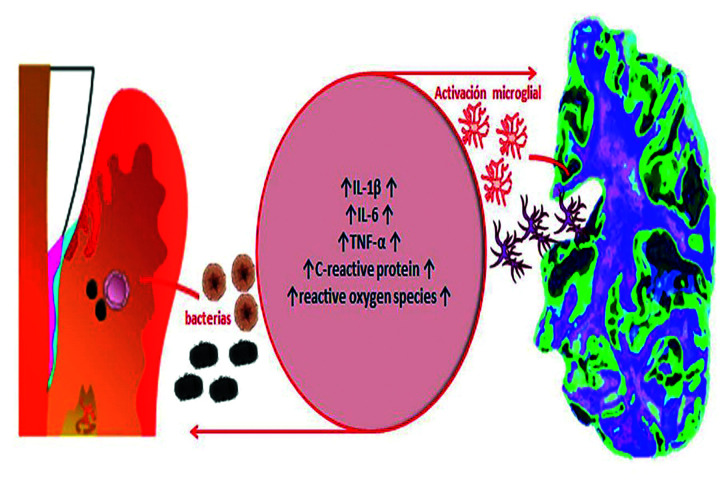
Common pathophysiological pathways between AD and periodontitis. The increase in inflammatory activity is the point of confluence between AD and periodontitis. As a result of the activity of periodontopathogens on the one hand and microglial activation on the other, an increase in the levels of the main proinflammatory molecules and their metabolites is produced, when this state is maintained in the long term it generates tissue destruction.

Similar pathological pathways have been reported in the establishment of AD and PE, including cellular and molecular characteristics such as oxidative damage and inflammation (Fig. 1). A model of association suggest that during the course of PE, locally produced pro-inflammatory cytokines diffuse from the ulcerated periodontal pocket into the systemic circulation around the trigeminal nerve terminals. Also, it has been suggested that bacteria or its virulence factors could spread from periodontal tissues to the brain through peripheric nerves, being the trigeminal nerve the main pathway. This molecules could induce neuroinflammation and, under chronic condition of neuroinflammation, mediators activated pro-inflammatory microglia phenotype, known as M1. (19-21).

**Figure 2:**
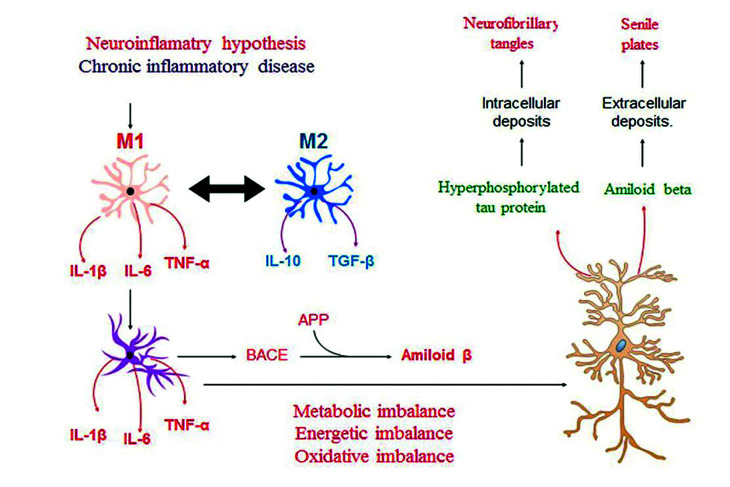
Neuroinflammatory hypotesis in the establishment of AD. When the inflammatory state is persistent, microglia and astrocytes modify their phenotype to reactive cells, increasing the secretion of pro-inflammatory cytokines and generating a metabolic, energetic and oxidative imbalance in the neuron that will respond by increasing the production of Aβ and the hyperphosphorylation of the microtubule-associated protein, Tau, events that generate neuro and synaptotoxicity.

In a state of health, microglia, the mononuclear phagocytes destined to limit injury in the central nervous system, maintain a neuroprotective function by eliminating Aβ peptides and expressing protective factors such as Insulin Growth Factor (IGF-1), Transforming Growth Factor -β and Nerve Growth Factor (22). Under inflammatory condition, the microglias differentiate toward a modulator phenotype or M2, which is characterized by secreting IL-10 and tumor growth factor (TGF)-β1. When this inflammatory state becomes persistent, the microglia modify its phenotype from the modulatory M2 to the pro-inflammatory M1. The M1 cells increase the production of IL-1β, IL-6 and TNF-α and are able to modify the function of astrocytes. The physiological astrocyte modifies its phenotype to a reactive astrocyte that responds by increasing the secretion of pro-inflammatory cytokines and the production of the amyloid precursor protein (APP) and of β and γ-secretases, generating the production of Aβ. The modification of the astrocyte function generates a metabolic, energetic and oxidative imbalance in the neuron that will respond by increasing the production of Aβ and the hyperphosphorylation of the microtubule-associated protein, Tau. These molecular events generate neuro and synaptotoxicity, constituting the main histopathological markers of AD (Fig. 2).

In general terms, microglia play a neuroprotective role, but in inflammatory conditions a neuroinflammatory role, thus being the central cell at the onset of AD (23).

The amyloid cascade hypothesis establishes Aβ as the central axis of neurodegeneration. Indeed, the Aβ peptide once secreted by astrocytes or neurons can undergo enzymatic modifications as non-enzymatic, generating its insolubilization. Insolubilization of Aβ peptides generate their accumulation in the brain and the establishment of senile plaques. Both soluble and insoluble Aβ are detected in the brains of patients affected by AD and, in this context, the presence of insoluble Aβ in the brain is directly correlated with the loss of synapse and cognitive impairment, being considered a marker of AD (24-26).

A neurodegenerative process secondary to the aggregation of Aβ in AD is the formation of NFTs, formed by a hyperphosphorylated form of the Tau protein that is expressed primarily in neurons. Both Aβ peptides and the immune response induced before Aβ peptides generate hyperphosphorylation of Tau proteins and the formation of NFTs and thus, axonal dysfunction that generates higher accumulation of Aβ in the synaptic spaces. In the initial stages of AD, these lesions are found in the entorhinal cortex and in the region of the dentate gyrus (GD) of the hippocampus —regions of the brain associated with memory processing and storage— and during the progression they extend from the GD to the field of Amón (CA)3, CA2 and CA1 to later affect the different areas of the cerebral cortex. The presence of these protein aggregates and dysfunctional proteins induce an immune-inflammatory response in the affected regions. Thus, the presence of pro-inflammatory molecules secreted by microglia, astrocytes, endothelial cells or neurons in response to these proteins, such as IL-1β and IL-6, TNF-α or INF-γ generate the vicious cycle of neuronal injury (27-29). Thus, NFTs are considered a true progression of AD marker, and insoluble Aβ, a marker for diagnosis.

- Role of the periodontal bacteria

**Figure 3:**
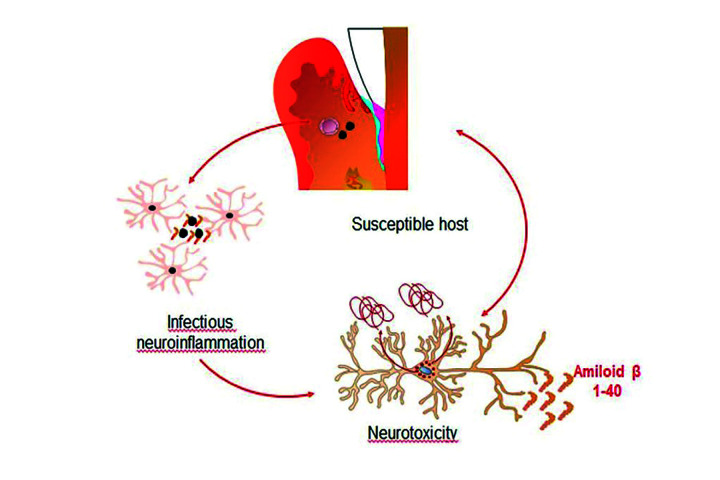
Inflammatory response of brain cells to oral bacteria. Model that suggests that periodontal bacteria and their products can invade the brain via and possibly generate a local inflammatory response and neurotoxicity.

In animal models it has been shown that chronic neuroinflammation induced by inoculation or injection of *Porphyromonas *gingivalis** Lipopolysaccharide (LPS) causes exacerbation in the production of pro-inflammatory cytokines and mediators which leads to hyperphosphorylation of Tau protein and an increase in Aβ levels contributing to neuronal deterioration (30,31). Numerous bacteria have been studied to determine a possible role of infection at the onset of AD. Thus, *Treponema denticola* and *Treponema pallidum* have been detected in the trigeminal ganglion and cortex and *Porphyromonas *gingivalis** in the fourth ventricle and cerebrospinal fluid (32,33). These data allow us to speculate that bacteria are able to invade the brain and possibly generate a local inflammatory response and neurotoxicity (Fig. 3) (33).

The mechanism of bacterial invasion to the brain are still unknown, however three models are proposed. The first establish that the bacteria spread into the bloodstream and, once in the cerebral vessels, the inflammatory response associated with cerebral vascular atherosclerosis could induce the breakdown of the blood-brain barrier (BBB). The second determine that oral bacteria would migrate through the peripheral terminations of the trigeminal nerve to the trigeminal ganglion and then, to the brain. The third route establishes that bacteria could migrate by lymphatic circulation. Indeed, the IV ventricle and the oral regional lymph nodes drain to a common lymph node, the mid-deep cervical lymph node (32,34). Although the three theories explain the possible routes of migration of oral bacteria to the brain, it has not yet been proven by which of them these bacteria can enter more easily.

- Periodontitis as a source of systemic inflammation.

One of the distinctive features of AD is the presence of activated glial cells that produce significant levels of inflammation. Suggesting that processes capable of regulating the expression of inflammatory molecules would contribute to their progression once established.

Pro-inflammatory molecules derived from the periphery could increase the set of inflammatory molecules in the brain by at least two mechanisms, through systemic circulation or peripheral nerve endings. Pro-inflammatory molecules in the systemic circulation could enter to the CNS in multiple ways. They could enter through areas of the brain that lack a BBB, but they could also enter in areas with BBB by various mechanisms: 1) diffusion through fenestrated capillaries of the BBB, 2) using specific cytokine transporters, 3) increasing the permeability of BBB or 4) activating brain endothelial cells to produce cytokines that induce signaling molecules such as nitrous oxide or prostanoids (35,36).

Once in the brain, these pro-inflammatory molecules increase the amount of cytokines, but also stimulate glial cells to synthesize additional pro-inflammatory cytokines (37). Several studies have determined that peripheral cytokines have the ability to stimulate afferent fibers of peripheral nerves, leading to increased levels of brain cytokines, or they may also have the ability to enter the brain through channels or compartments associated with peripheral nerves (36,37). The last hypothesis suggests that pro-inflammatory molecules originating in the oral cavity can reach the brain through neurological pathways (37).

Additionally, bacterial products or bacteria themselves can increase the concentration of brain cytokines. Various bacteria have been identified as cofactors in the etiology and pathogenesis of AD (22), including *Chlamydia Pneumoniae*, *Borrelia burgdoferi* and *Treponema denticola*. Similarly, the findings indicate that periodontal bacteria, *Aggregatibacter actinomycetemcomitans*, *Porphyromonas *gingivalis**, *Prevotella intermedia*, *Fusobacterium nucleatum*, *Tannerella forsythia*, *Eikenella corrodens* and T. denticola, can invade the brain through two pathways: through bloodstream or through peripheral nerves (38,39).

Finally, a bidirectional relationship between both diseases has also been proposed, there being a coherent relationship so that AD can predispose to the development of PE, this is explained because patients with AD have a worse oral hygiene, either because their dexterity manual to carry out daily oral hygiene is diminished or absent or due to the inability of the subject himself to go to the dentist for professional care (40).

## Discussion

This review of the literature contributes to the understanding of the pathological pathways that both diseases share. There is still not enough evidence to determine an association between the two pathologies, however the available evidence indicates a positive trend towards the association between them. The existence of a causal relationship between them is unknown due to the heterogeneity of the designs used. For this reason, it is considered necessary to carry out studies to determine if PE is capable of inducing or exacerbating the neuroinflammation that will trigger AD.
